# Indirect effects of health-related quality of life on suicidal ideation through psychological distress among cancer patients

**DOI:** 10.1177/13591053231225306

**Published:** 2024-01-26

**Authors:** Nkechi A Chukwuemeka, Tosin Yinka Akintunde, Favour E Uzoigwe, Marvellous Okeke, Andrew Tassang, Stanley Oloji Isangha

**Affiliations:** 1 University of Nigeria Nsukka, Nigeria; 2 Hohai University, China; 3 Chinese University of Hong Kong, Hong Kong; 4 Southeast University School of Medicine, China; 5 University of Buea, Cameroon; 6 Buea Regional Hospital, Annex, Cameroon; 7 City University of Hong Kong, Hong Kong

**Keywords:** cancer, health-related quality of life, psychological distress, suicidal ideation

## Abstract

The interrelationships of suicidal ideation, psychological distress, and impaired health-related quality of life (HRQoL) in cancer patients are complex and multifaceted. Limited empirical evidence exists on the indirect effects of impaired HRQoL on suicidal ideation through psychological distress among cancer patients. To fill this research gap, 250 cancer patients were recruited through a cross-sectional hospital-based research design. Structural equation model (SEM) results indicated that impaired HRQoL is a predictor of psychological distress (β = 0.153; *p* < 0.05), and psychological distress positively predicts suicidal ideation (β = 0.647; *p* < 0.000). The study found no direct effects of impaired HRQoL on suicidal ideation (β = −0.05; *p* = 0.223). Indirect effects of HRQoL on suicidal ideation was confirmed, showing a full-mediation effect β = 0.099 (SE = 0.048, CI = [0.030, 0.189], *p* < 0.05) (i.e. the pathway impaired HRQoL predict suicidal ideation is through psychological distress). Cognitive-behavioral therapy and other emotional support programs should be considered for cancer patients to mitigate psychological vulnerabilities linking impaired HRQoL to suicidal ideation.

## Introduction

More than 800,000 suicides occur yearly, making it the second leading cause of death among those aged 15–29 years old (WHO, 2019). In recent years, extensive research has been conducted on suicide among cancer patients as a serious public health concern (Anguiano et al., 2012; Du et al., 2020; Musa et al., 2021, 2022). As a debilitating health condition, cancer disrupts physical, mental, and social functioning, leaving survivors with unmet needs (Fang et al., 2018; Silvaggi et al., 2023; Yang et al., 2023). Adverse effects of cancer are typically accompanied by multimorbidity, such as psychological distress and suicidal ideation. Suicidal ideation is the process of mulling over killing oneself (Klonsky et al., 2016). It may range from fleeting thoughts to detailed planning, depending on the severity of the mental illness (Bryan et al., 2019). The odds of suicidal ideation become higher with increasing psychological problems among cancer patients (Peltzer and Pengpid, 2022). Currently, suicidal ideation poses significant policy and intervention challenges (Desalegn et al., 2020; WHO, 2019).

The assessment of suicidal ideation is not a simple process since no single factor determines the likelihood of a completed suicide. Several risk factors for suicide have been identified among the general population, including a history of self-harm or attempts, mental health diagnosis, psychological distress, and impaired health-related quality of life (HRQoL) (CDC, 2018; Lopez-Castroman et al., 2020; Twenge et al., 2018). Notably, suicidal ideation is a significant precursor to suicide among cancer patients, with prevalence ranging from 1.1% to 19.8% (Casey et al., 2008). Despite reports that cancer patients are at risk of suicide (Anguiano et al., 2012; Du et al., 2020), not all cancer patients report suicidal ideation. Demoralization, financial burden, living conditions, and cancer types are found to contribute to suicidal ideation among cancer patients (Lai et al., 2022). Due to a lack of data on these psychosocial indicators, it is increasingly imperative to examine risk factors for suicidal ideation in cancer patients, especially those in resource-limited populations (Amiri and Behnezhad, 2020; Tang et al., 2022). Having such data is essential to educating clinicians and researchers regarding the risk factors and indirect mechanisms associated with suicidal ideation in cancer patients.

HRQoL is a multidimensional construct encompassing an individual’s physical, psychological, and social condition (Megari, 2013). Several studies have assessed the HRQoL of patients with various typologies of cancer (e.g., Ho et al., 2021; Lin et al., 2017; Wu et al., 2023). Suicidal ideation is known to be exacerbated by impaired HRQoL in cancer patients (van Oers and Schlebusch, 2020). Moreover, psychological distress can also lead to suicidal ideation (Lai et al., 2022). Psychological distress is characterized by depression, anxiety, and stress (Keles et al., 2020), existing as psychopathological comorbidities among cancer patients (Koné and Scharf, 2021; Naser et al., 2021). Psychological distress is more likely to occur in cancer patients with impaired HRQoL (Chan et al., 2014; Halvorsen et al., 2018). Suicidal ideation in cancer patients is further exacerbated by the interrelationships between HRQoL and psychological distress (Phoosuwan and Lundberg, 2022). Until now, the indirect effects of impaired HRQoL on suicidal ideation through psychological distress are generally less discussed among cancer patients, especially in resource-limited populations. Cancer patients from low-income countries are faced with socioeconomic disadvantages, poor healthcare infrastructure, and limited psychological interventions that could buffer suicidal ideation risks or even promote HRQoL (Adejoh et al., 2013). This study extends empirical evidence of HRQoL’s indirect effect on suicidal ideation through psychological distress using data from cancer patients.

### Literature and theoretical review

Studies have examined the relationship between HRQoL and suicidal ideation in cancer patients (Anguiano et al., 2012; Du et al., 2020). However, empirical investigations have not comprehensively examined the theoretical basis for this relationship. The psychological theory of learned helplessness (Maier and Seligman, 2016), provides a premise for understanding the process of these associations. Consistent focus on uncontrollable adverse circumstances (i.e. impaired HRQoL) can result in loss of control, leading to unmanageable and errant thought patterns (Lennerlöf, 1991). Cancer patients may face the reality of their deteriorating health with trepidation until they lose control of their emotions, leading to unstable thought patterns such as suicidal ideation. Psychological reactance models also reflect these psychosocial conflicts (Rosenberg and Siegel, 2018). Psychological reactance may occur when cancer patients experience unpleasant motivation as a result of their disease condition, which arises from the loss of autonomy over their health. If their health does not improve, they may become frustrated and develop adverse psychological reactions (De Las Cuevas et al., 2014). Cancer patients’ deteriorating HRQoL may increase their risk of suicidal ideation as their health autonomy declines. There is evidence that poor HRQoL is linked to suicidal ideation (van Oers and Schlebusch, 2020).

The link between HRQoL and psychological distress has also been verified in several empirical studies (Chan et al., 2014; Halvorsen et al., 2018). Based on the transactional model of stress and coping, individuals utilize cognitive and behavioral responses to deal with internal or external situations that exceed their ability to cope and adjust (Folkman and Lazarus, 1980). Psychological distress and HRQoL are determined by a transactional process, which includes stressors associated with cancer diagnosis, side effects of treatment, and frailty. The effects of these stressors can result in a subdued emotional adjustment and a further suppression of cognitive functioning (Baik et al., 2020). Cognitive-behavioral theory clarifies the transactional processes between impaired HRQoL and psychological distress in cancer patients (Lai et al., 2019). Thoughts and thinking patterns can contribute to psychological distress (González-Prendes and Resko, 2012). Negative thinking patterns associated with impaired HRQoL may lead to adverse expectations that elevate distress levels. Cancer patients’ psychological distress is significantly influenced by their compromised health status (Phoosuwan and Lundberg, 2022).

Meanwhile, through the process of emotional regulation (Saarni et al., 1998), cancer patients may experience suicidal ideation arising from the difficulty in controlling their exposure to psychological vulnerabilities. In a normal, nonmorbid individual, emotions may be controlled based on limited adverse external or internal influences that could promote deficits in mental health and behavior (Thompson, 1991). The presence of unstable and problematic emotions, such as depression, anxiety, or stress, could indicate a dysfunctional emotional state in cancer patients. Emotional dysfunction, such as excessive burden and worry, can result in emotional distress and suicidal ideation (Thullen et al., 2016). Suicidal ideation is further aggravated by emotional distortions, such as losing meaning and purpose in life (Hirsch et al., 2017).

These assertions discussed above may explain the direct association among HRQoL, psychological distress, and suicidal ideation. However, the understanding of the indirect mechanism warrants further theoretical exploration. The interrelationships of impaired HRQoL, psychological distress, and suicidal ideation are based on psychopathology schemas arising from adversity, such as cancer (Ingram and Luxton, 2005). According to stress-diathesis models of suicidal behavior, adversity components, such as impaired HRQoL could motivate self-harm behavior (van Heeringen, 2012). The inability to cope with or control stresses or adversity contributes to psychological distress (Mann and Haghighi, 2010). When HRQoL is compromised in cancer patients, distress may be aggravated, resulting in suicidal ideation. Postulations from the dual-process model of illness suggest that individual susceptibilities (e.g. psychological distress) and stressors (e.g. impaired HRQoL) could contribute to suicidal ideation (Berrenberg, 1991). Two psychosocial factors are combined and interact to result in an adverse outcome in the dual-process model of illness (Pyszczynski et al., 1999). For instance, deteriorating HRQoL and psychological distress could work in tandem through two distinct processes. Among the factors that influence suicidal ideation, psychological distress may be the most immediate and proximal (Ensel et al., 1996), whereas impaired HRQoL is considered a more distal factor (Murdaugh, 1997). Thus, impaired HRQoL may indirectly contribute to suicidal ideation by increasing psychological distress among cancer patients. Through these theoretical propositions, this study examined;

i. the direct effects of impaired HRQoL on psychological distress and suicidal ideation
ii. the direct effect of psychological distress on suicidal ideation
iii. and indirect effects of impaired HRQoL on suicidal ideation through psychological distress

## Methods

Using a cross-sectional hospital-based research design, 250 patients were enrolled in this study at the University of Nigeria Teaching Hospital, Enugu, Nigeria. This study was conducted between March 2023 and July 2023 using a purposive sampling. Nurses and trained enumerators administered questionnaires during appointment days in the outpatient care unit. On days of appointment in the hospital’s oncology ward, the nurses introduced the trained enumerators and explained the purpose and significance of the study. Only patients in the early cancer stages, that is, in stage I and II, were included in this study. Out of about 280 potential participants invited for the survey, approximately 11% declined participation. The University of Nigeria Teaching Hospital Health Research Ethics Committee provided an ethical approval letter for the study (UNTH/HREC/2023/01/511) on January 18th, 2023. All participants participated voluntarily and were assured of anonymity and confidentiality of all information provided.

### Measures

#### Suicidal ideation

The suicidal ideation attribute scale (SIDAS) was used to measure suicidal ideation severity (van Spijker et al., 2014). SIDAS is a 5-item scale assessing frequency (item 1), controllability (item 2), closeness to attempt (item 3), distress (item 4), and interference with daily activities (item 5) on 10-point scale over the past month ranging from 0 to 10. SIDAS was validated by correlating it with the two scales of the emotion regulation questionnaire (Amazue et al., 2019). Amazue et al. (2019) also reported the internal consistency reliability coefficient of Cronbach α = 0.94 for SIDAS. SIDAs has a Cronbach alpha of 0.86 in this study. The prevalence among the participants based on the score estimates in Supplemental Table 2 indicated no suicidal ideation = 0.4%, low suicidal ideation = 89.6, and high suicidal ideation = 8.8%.

#### Health-related quality of life (HRQoL)

HRQoL was measured using the Functional Assessment of Cancer Therapy-General (FACT-G) developed by Cella et al. (1993). The FACT-G is a self-report measure with a 5-point Likert response format with a rating scale (0 = Not at all; 1 = A little bit; 2 = Somewhat; 3 = Quite a bit; and 4 = Very much) (Cella et al., 1993). The scale is divided into four sub-scales: Physical wellbeing (PWB; seven items, score range from 0 to 28), social/family wellbeing (SFWB; 7-items, score range from 0 to 28), emotional wellbeing (EWB; 6-items, score range from 0 to 24), and functional wellbeing (FWB; 7-items, score range from 0 to 28). Two subscales, that is, SFWB and FWB, were reverse-coded. Cella et al. (1993) indicated that the Cronbach’s alpha of the total scale is 0.89.

#### Psychological distress

The Depression Anxiety Stress Scales (DASS-21) was designed to measure the core symptoms of depression, anxiety, and stress and has demonstrated excellent psychometric properties across studies mostly conducted in Western societies (Lovibond and Lovibond, 1995). DASS-21 is a 21-item standardized instrument developed as a shorter version of DASS-42. It is an instrument that assesses depression, anxiety, and stress levels of individuals. Each item was rated on a 4-point Likert scale from 0, meaning “did not apply to me at all,” to 3 “applied to me very much or most of the time.” The convergent validity of DASS-21 has been found to have a positive relationship with the Beck Depression Inventory between the three subscales (Asghari et al., 2018). Among age-specific populations, the DASS-21 acceptable factor loading ranges from 0.76 to 0.92 across different times and subscales (Cao et al., 2023a, 2023b; Chen et al., 2023). For the total score of DASS-21, the Cronbach alpha = 0.94. The Cronbach alpha for Depression, Anxiety, and Stress scales were 0.85, 0.85, and 0.87, respectively. Prevalence of psychological distress among the participant as reported in Supplemental Figure 1 shows that; Depression: normal = 56.8%, mild-moderate = 36%, and severe-extremely severe = 7.2%; Stress: normal = 76.8%, mild-moderate = 20.4%, and severe-extremely severe = 2.8%; Anxiety: normal = 12.4%, mild-moderate = 56.8%, and severe-extremely severe = 30.8%.

#### Control variables

In the model, age, cancer types, and level of education were controlled (see Supplemental Table 1).

##### Analysis

Descriptive analysis of the study was done through bivariate analysis (correlations), mean and standard deviations were computed through the Statistical Package for Social Sciences (SPSS Version 25.0). In addition, AMOS Version 20 was used to analyze the latent and observed variables through maximum likelihood analysis based on the structural equation model (SEM). As part of the test of the SEM model in the dataset, we examined the model’s goodness of fit through confirmatory factor analysis (Bentler, 1990). A bootstrapping method was used to investigate the indirect effect of psychological distress in the theoretical model (*n* = 5000 bootstrapping randomly selected samples) (Preacher and Hayes, 2008). To verify the SEM model fit, we used Comparative Fit Index (CFI), Incremental Fit Index (IFI), and Normed Fit Index (NFI) benchmarked at 0.95, and the root means square error of approximation (RMSEA) acceptable when below 0.06 (Bentler et al., 1987; Hu and Bentler, 1999). Impaired HRQoL was measured by scoring the four subscales as an observed variable. Two subscales (SFWB and FWB) of the HRQoL were recoded to allow the observed variable to indicate impaired HRQoL across the sub-scales. Psychological distress was measured as a latent variable with factor loading anxiety = 0.52, stress = 0.42, and depression = 0.88. The factor loading of the four items retained of suicidal ideation ranges from 0.53 to 1.00. In addition, to resolve the multicollinearity issues concerning measurements of HRQoL and psychological distress, a variance-inflated factor (VIF) analysis was conducted (Jung and Park, 2018). The VIF assumes accepting that the predictors have no multicollinearity if VIF values are less than 5 as detailed in Table 1 (Marcoulides and Raykov, 2019). The pre-analysis results indicate excellent model fit for the data (χ^2^(18, *N* = 250) = 25.985, *p* > 0.100; CMIN/DF = 1.444, and with NFI = 0.979, TLI = 0.990, CFI = 0.993, and RMSEA = 0.040).

**Table 1.** Mean, SD, and correlation matrix of the study measures.

	Mean (SD)	VIF	1	2	3	4	5	6	7	8	9	10	11
1. Suicidal ideation	10.96 (3.605)	—	1										
2. Stress	3.58 (2.080)	1.388	0.244**	1									
3. Depression	3.74 (2.926)	1.569	0.635**	0.389**	1								
4. Anxiety	1.28 (1.393)	1.276	0.421**	0.180**	0.448**	1							
5. PWB	5.63 (1.629)	1.155	0.065	−0.078	0.142*	−0.003	1						
6. EWB	8.80 (4.390)	1.317	0.206**	0.380**	0.217**	0.001	0.193**	1					
7. SWB	3.39 (2.262)	1.145	0.213**	0.180**	0.262**	0.160*	0.081	0.255**	1				
8. FWB	13.83 (4.675)	1.124	−0.249**	−0.068	−0.188**	−0.009	−0.239**	−0.158*	0.046	1			
9. Age-group			−0.134*	0.046	−0.180**	−0.112	0.042	−0.034	−0.234**	0.021	1		
10. Education			−0.010	−0.083	0.001	0.054	0.091	−0.071	0.154*	0.126*	0.025	1	
11. Cancer types			0.529**	0.241**	0.344**	0.108	0.251**	0.284**	0.018	−0.384**	0.055	−0.068	1

1 = reference value for comparative variables.

VIF: variance inflation factors (multicollinearity criteria).

* *p* < 0.05. **p < 0.01.

## Result

Data from 250 cancer patients were analyzed. The data distribution includes 73.6% females and 26.4% males. Other information on the socioeconomic and demographic characteristics of the study population is shown in Supplemental Table 1.

The mean, standard deviation, and correlation for the study variables are shown in Table 1. The VIF result indicates that the variables are not multicollinear. Correlations among all the measured variables indicate that suicidal ideation is positively correlated with psychological distress indicators (i.e. stress (*r* = 0.244, *p* < 0.01), depression (*r* = 0.635, *p* < 0.01), and anxiety (*r* = 0.421, *p* < 0.01)). In addition, suicidal ideation is positively correlated with EWB (*r* = 0.206, *p* < 0.01) and SFWB (*r* = 0.231, *p* < 0.01). However, suicidal ideation is negatively associated with FWB (*r* = −0.249, *p* < 0.01). The outcome based on the bivariate exploration of socioeconomic and demographic variables indicated that age is negatively correlated with suicidal ideation (*r* = −0.134, *p* < 0.05), while cancer types were positively associated with suicidal ideation (*r* = 0.529, *p* < 0.01).

### Main model

**Figure 1.**
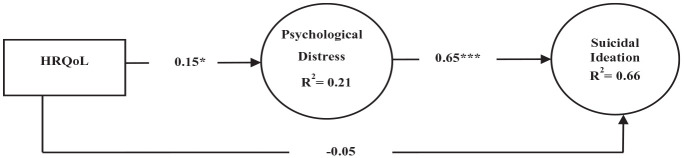
Structural equation model (SEM) of direct and indirect effects on suicidal ideation for the total population. Controlled for age (β = −0.200; *p* < 0.01), education (not significant), and cancer types (β = 0.458; p < 0.001). HRQoL: health-related quality of life. *p < 0.05 .**p < 0.01 .***p < 0.001.

The analysis results premised on the total population provided a good fit for the data (χ^2^(36, *N* = 250) = 55.631, *p* < 0.05, and with NFI = 0.959, TLI = 0.977, CFI = 0.985, and RMSEA = 0.047). Figure 1 shows the path outcome in the model such that impaired HRQoL was positively associated with psychological distress (β = 0.153; *p* < 0.05), indicating that when HRQoL worsens by 1 standard deviation, psychological distress goes up by 0.153 standard deviation. Psychological distress is positively associated with suicidal ideation (β = 0.647; *p* < 0.001), indicating that with 1 unit standard deviation increase in psychological distress, suicideal ideation goes up by 0.647 standard deviation. Overall, analysis shows that the model explained 21.2% of the variance in psychological distress. In addition, a 66% variance in suicidal ideation in the cancer patients was explained by the model.

To verify the indirect relationships in the theoretical model, we generated 5000 samples (through a bootstrapping sampling strategy) from the primary dataset by random sampling. The result in Table 2 shows that the indirect effects of impaired HRQoL on suicidal ideation through psychological distress was β = 0.099 (SE = 0.048, CI = [0.030, 0.189], *p* < 0.05). This result indicated that the standardized indirect effect of impaired HRQoL on suicidal ideation is 0.099 (i.e. when HRQoL worsens by 1 standard deviation, suicidal ideation goes up by 0.099 standard deviations). These results consolidate the understanding that the indirect pathway through which impaired HRQoL could result in suicidal ideation among cancer patient is through psychological distress.

**Table 2.** Indirect effect of HRQL on suicidal ideation through psychological distress.

	Effect size	SE	LB-UB [95% CI]	*p*-Value
Total effect	0.045	0.046	[−0.029, 0.121]	0.296
Direct effect	−0.054	0.046	[−0.130, 0.022]	0.232
Indirect effect	0.099	0.048	[0.030, 0.189]	**0.05**

Bold: Significant at p < 0.05 LB: lower bound; UB: upper bound; SE: standard error; CI: confidence interval.

## Discussion

This study examined impaired HRQoL’s direct effects on psychological distress and suicidal ideation. The study extends the theoretical premises of stress-diathesis and the dual-process model of illness by investigating the indirect effects of impaired HRQoL on suicidal ideation through psychological distress among cancer patients.

The finding shows no direct and significant association between HRQoL and suicidal ideation among the cancer patients. This finding is counterintuitive to studies and theories relating to psychological reactance and learned helplessness supporting the effects of impaired HRQoL on suicidal ideation (Lennerlöf, 1991; Rosenberg and Siegel, 2018; van Oers and Schlebusch, 2020). Several subjective and cultural factors may account for these discrepancies in the findings and theoretical understanding of the psychopathology. Based on the sociocultural characteristics of this study population, patients may have access to alternative coping strategies, such as religious and spiritual beliefs, which may prevent suicidal ideation regardless of HRQoL impairment (Kelly et al., 2022; Mabena and Moodley, 2012). These factors could also lead to a reluctance to discuss suicidal thoughts due to stigmatization or faith-based assurance (Bruce et al., 2020; Okyere et al., 2022). Hence, the study cannot confirm that impaired HRQoL and suicidal ideation are associated. This finding confirms that impaired HRQoL is associated with suicidal ideation as a function of the socio-environment and population.

However, the study found that impairment of HRQoL was a positive predictor of psychological distress in cancer patients. Earlier studies and theories have suggested that stressors (such as diagnosis and treatment-related side effects) may adversely affect the psychological wellbeing of cancer patients (Baik et al., 2020). The study also identified other direct paths, suggesting that psychological distress is positively associated with suicidal ideation. According to previous theories and studies of emotional regulation (Saarni et al., 1998), cancer patients may experience instability and problematic emotions, such as fear, sadness, and anger, leading to thoughts of suicide. Symptoms of these emotional dysfunctions, such as anxiety, stress, and depression, can lead to suicidal thoughts. Consequently, impaired HRQoL could promote psychological distress, and psychological distress is a risk factor for suicidal ideation among cancer patients.

Furthermore, impaired HRQoL indirectly contributes to suicidal ideation through psychological distress. This study indicates that two theoretical propositions (the dual process model of illness and the stress-diathesis model) are viable theoretical perspectives that could be used to understand the indirect link between impaired HRQoL and suicidal ideation through psychological distress (Pyszczynski et al., 1999; van Heeringen, 2012). According to the indirect pathway, impaired HRQoL and psychological distress may interact to exacerbate suicidal ideation in cancer patients (Ensel et al., 1996; Murdaugh, 1997), and together they form psychopathology schemas in which impaired HRQoL leads to psychological distress, and psychological distress leads to suicidal ideation. Based on the study findings, cancer patients’ suicidal ideation may not be directly caused by impaired HRQoL but rather indrectly through psychological distress, leading to a far-reaching adverse outcome.

Age, cancer types, and educational attainment of the cancer patients were also controlled for in this study’s theoretical model, which may have contributed to the results. According to the model, 21% of psychological distress variance is predicted. However, more research is needed to examine psychosocial factors that might contribute to the unexplained variance in psychological distress among cancer patients examined in this study within their unique sociocultural context. Suicidal ideation accounts for 66% of the variance explained by this model. Suicidal ideation may be cushioned by urgent HRQoL and psychological distress interventions for this unique population.

Despite its theoretical and empirical merits, this study has limitations. The relatively small sample size (250 respondents) and the study setting (limited to only University of Nigeria Teaching Hospital, Enugu, Nigeria) seem to limit the generalizability of this finding. Data collection should be expanded to other regions in Nigeria in future studies. A comparative study of the indirect mechanism proposed in this study is also encouraged. Additionally, this study was cross-sectional with self-report measures, so causal inferences cannot be drawn. Future studies should adopt a longitudinal approach, which may help determine causality. Research replicating the research objectives should control for treatment and lengths of treatments to extend this study.

### Theoretical and practical implication

Certain populations, especially those from specific sociocultural and environmental backgrounds, are less likely to report suicidal ideation when HRQoL is impaired. Suicidal ideation and impaired HRQoL are not associative due to numerous coping mechanisms available to cancer patients, including spirituality, religion, and social desirability (Bruce et al., 2020; Okyere et al., 2022). Providing emotional support for cancer patients should take precedence on their road to recovery. Psychological reactions and learned helplessness may not hold in specific psychosocial contexts or among some populations, as critically ill individuals may seek coping mechanisms to arrest suicidal ideation in the face of impaired HRQoL.

In parallel with ongoing debates on the influences of psychological distress on the association between HRQoL and suicidal ideation among cancer patients, the study advances empirical evidence. Cancer patients can benefit from psychological distress buffering resources like mindfulness, cognitive-behavioral therapies, and counseling programs by arresting these negative interactions of HRQoL, psychological distress, and suicidal ideation. This approach is necessary because psychological distress is one pathway through which cancer patients with deteriorating HRQoL may experience suicidal ideation. Suspending exposure to emotional vulnerabilities becomes a viable intervention option for clinicians and public health policies to cushion the effects of impaired HRQoL on suicidal ideation. Clinicians, the government, and public health stakeholders must work together to provide appropriate policies, cognitive behavioral therapy, and other psychosocial interventions.

## Conclusion

Based on the results of this study, impaired HRQoL was associated with psychological distress. Suicide ideation is also significantly influenced by psychological distress. There was no direct effect of impaired HRQoL on suicidal ideation among cancer patients. This research evidence suggests that HRQoL affects suicidal ideation indirectly through psychological distress, as indicated by the full mediation effect of the study model. Psychological vulnerabilities of cancer patients should be mitigated through cognitive-behavioral therapies and other programs to cushion the adverse effects.

## Supplemental Material

sj-docx-1-hpq-10.1177_13591053231225306 – Supplemental material for Indirect effects of health-related quality of life on suicidal ideation through psychological distress among cancer patients Supplemental material, sj-docx-1-hpq-10.1177_13591053231225306 for Indirect effects of health-related quality of life on suicidal ideation through psychological distress among cancer patients by Nkechi A Chukwuemeka, Tosin Yinka Akintunde, Favour E Uzoigwe, Marvellous Okeke, Andrew Tassang and Stanley Oloji Isangha in Journal of Health Psychology

sj-docx-2-hpq-10.1177_13591053231225306 – Supplemental material for Indirect effects of health-related quality of life on suicidal ideation through psychological distress among cancer patients Supplemental material, sj-docx-2-hpq-10.1177_13591053231225306 for Indirect effects of health-related quality of life on suicidal ideation through psychological distress among cancer patients by Nkechi A Chukwuemeka, Tosin Yinka Akintunde, Favour E Uzoigwe, Marvellous Okeke, Andrew Tassang and Stanley Oloji Isangha in Journal of Health Psychology

sj-docx-3-hpq-10.1177_13591053231225306 – Supplemental material for Indirect effects of health-related quality of life on suicidal ideation through psychological distress among cancer patients Supplemental material, sj-docx-3-hpq-10.1177_13591053231225306 for Indirect effects of health-related quality of life on suicidal ideation through psychological distress among cancer patients by Nkechi A Chukwuemeka, Tosin Yinka Akintunde, Favour E Uzoigwe, Marvellous Okeke, Andrew Tassang and Stanley Oloji Isangha in Journal of Health Psychology
