# Supplementary material for: Indirect effects of health-related quality of life on suicidal ideation through psychological distress among cancer patients
Source: J Health Psychol. 2024 Jan 26;29(10):1061–73. doi: 10.1177/13591053231225306 (PMC11344958; doi:10.1177/13591053231225306)
Supplement: sj-docx-1-hpq-10.1177_13591053231225306 – Supplemental material for Indirect effects of health-related quality of life on suicidal ideation through psychological distress among cancer patients [file sj-docx-1-hpq-10.1177_13591053231225306.docx]

Supplementary Figure 1. Psychological Distress Score
