# Supplementary material for: Indirect effects of health-related quality of life on suicidal ideation through psychological distress among cancer patients
Source: J Health Psychol. 2024 Jan 26;29(10):1061–73. doi: 10.1177/13591053231225306 (PMC11344958; doi:10.1177/13591053231225306)
Supplement: sj-docx-2-hpq-10.1177_13591053231225306 – Supplemental material for Indirect effects of health-related quality of life on suicidal ideation through psychological distress among cancer patients [file sj-docx-2-hpq-10.1177_13591053231225306.docx]

Supplementary Table 1. Socioeconomic attributes and and cancer types, mean and standard deviation of variables (Total = 250)

| Variables | Total (%) | Mean (SD) |
| --- | --- | --- |
| **Age** |  | 3.6(1.4) |
| ≤25 | 6 (2.4) |  |
| 26-35 | 51(20.4) |  |
| 36-45 | 79(31.6) |  |
| 46-55 | 50(20.0) |  |
| 56-65 | 32(12.8) |  |
| ≥66 | 32(12.8) |  |
| **Gender** |  | 1.3(0.4) |
| Female | 184(73.6) |  |
| Male | 66 (26.4) |  |
| **Education Level** |  | 2.6(0.7) |
| None | 5(2) |  |
| Elementary/Primary | 26(10.4) |  |
| Secondary/Highschool | 63(25.2) |  |
| University/ Postgraduate | 156(62.4 |  |
| **Marital Status** |  | 1.8(0.4) |
| Single | 48(19.2) |  |
| Married | 292(80.8) |  |
| **Employment** |  | 1.4(0.5) |
| Employed | 151(60.4 |  |
| Unemployed | 99(39.6) |  |
| **Cancer Types** |  | 1.9(0.9) |
| Prostrate | 75(30.0) |  |
| Breast | 146(58.4) |  |
| Lung | 9(3.6) |  |
| Skin | 10(4.0) |  |
| Others (i.e., colon, rectal e.t.c) | 10(4.0) |  |
