# Supplementary material for: Indirect effects of health-related quality of life on suicidal ideation through psychological distress among cancer patients
Source: J Health Psychol. 2024 Jan 26;29(10):1061–73. doi: 10.1177/13591053231225306 (PMC11344958; doi:10.1177/13591053231225306)
Supplement: sj-docx-3-hpq-10.1177_13591053231225306 – Supplemental material for Indirect effects of health-related quality of life on suicidal ideation through psychological distress among cancer patients [file sj-docx-3-hpq-10.1177_13591053231225306.docx]

Supplementary Table 2. Prevalence of Suicide Ideation

| **Prevalence** | **Frequency (%)** |
| --- | --- |
| No Ideation | 1(0.4) |
| Low Ideation | 224(89.6) |
| High Ideation | 22(8.8) |

Non-response: 3(1.2%)
